# Discovery of new drug indications for COVID-19: A drug repurposing approach

**DOI:** 10.1371/journal.pone.0267095

**Published:** 2022-05-24

**Authors:** Priyanka Kumari, Bikram Pradhan, Maria Koromina, George P. Patrinos, Kristel Van Steen

**Affiliations:** 1 GIGA-R Medical Genomics - BIO3 Systems Genomics, University of Liège, Liège, Belgium; 2 Laboratory of Pharmaceutical Analytical Chemistry, CIRM, University of Liège, Liège, Belgium; 3 Indian Space Research Organisation (ISRO) Headquarters, Bengaluru, India; 4 University of Patras, School of Health Sciences, Department of Pharmacy, Patras, Greece; 5 Department of Pathology, College of Medicine and Health Sciences, United Arab Emirates University, Al Ain, UAE; 6 Zayed Bin Sultan Center for Health Sciences, United Arab Emirates University, Al Ain, UAE; 7 Department of Human Genetics - BIO3 Systems Medicine, University of Leuven, Leuven, Belgium; National Chiao Tung University College of Biological Science and Technology, TAIWAN

## Abstract

**Motivation:**

The outbreak of coronavirus health issues caused by COVID-19(SARS-CoV-2) creates a global threat to public health. Therefore, there is a need for effective remedial measures using existing and approved therapies with proven safety measures has several advantages. Dexamethasone (Pubchem ID: CID0000005743), baricitinib(Pubchem ID: CID44205240), remdesivir (PubchemID: CID121304016) are three generic drugs that have demonstrated *in-vitro* high antiviral activity against SARS-CoV-2. The present study aims to widen the search and explore the anti-SARS-CoV-2 properties of these potential drugs while looking for new drug indications with optimised benefits via *in-silico* research.

**Method:**

Here, we designed a unique drug-similarity model to repurpose existing drugs against SARS-CoV-2, using the anti-Covid properties of dexamethasone, baricitinib, and remdesivir as references. Known chemical-chemical interactions of reference drugs help extract interactive compounds withimprovedanti-SARS-CoV-2 properties. Here, we calculated the likelihood of these drug compounds treating SARS-CoV-2 related symptoms using chemical-protein interactions between the interactive compounds of the reference drugs and SARS-CoV-2 target genes. In particular, we adopted a two-tier clustering approach to generate a drug similarity model for the final selection of potential anti-SARS-CoV-2 drug molecules. Tier-1 clustering was based on t-Distributed Stochastic Neighbor Embedding (t-SNE) and aimed to filter and discard outlier drugs. The tier-2 analysis incorporated two cluster analyses performed in parallel using Ordering Points To Identify the Clustering Structure (OPTICS) and Hierarchical Agglomerative Clustering (HAC). As a result, itidentified clusters of drugs with similar actions. In addition, we carried out a docking study for *in-silico* validation of top candidate drugs.

**Result:**

Our drug similarity model highlighted ten drugs, including reference drugs that can act as potential therapeutics against SARS-CoV-2. The docking results suggested that doxorubicin showed the least binding energy compared to reference drugs. Their practical utility as anti-SARS-CoV-2 drugs, either individually or in combination, warrants further investigation.

## Introduction

Coronavirus (SARS-CoV-2) outbreaks, the first of which began in November/December 2019, have left only a few countries uninfected. However, the number of daily cases is constantly fluctuating at a global level. So far, there is no permanent cure nor a specific vaccine available that could potentially eliminate the adverse effects of SARS-CoV-2 infection. There is an urgent need to find effective and safe preventive medications which are easily accessible and inexpensive so that everyone can afford them. Repurposing already FDA-approved drugs or the drugs which are under clinical trials against SARS-CoV-2 can be a promising solution [1]: effective, cheap and time-efficientmethodology [2]. *In-silico* methods offer a way to systematically and rapidly yield drug repurposing candidates. The main hypothesis of drug repurposing is that there is a high possibility that a drug may have multiple protein targets and shared molecular pathways between diseases [3]. Dexamethasone, baricitinib and remdesivir are three FDA approved drugs that showed some positive signs against this deadly disease [4, 5] and helped in relieving SARS-CoV-2 related symptoms from patients across various countries. To this end, we decided to assess these three drugs in the present study for drug repurposing against SARS-CoV-2. These drugs are effective and mostly used for the initial treatment ofcovid-19 related issues. To our knowledge, it is the first time that dexamethasone, baricitinib and remdesivir have been studied together and used for repurposing against SARS-CoV-2 by FDA approval [6]. Baricitinib is an approved drug for rheumatoid arthritis(RA), and remdesivir is an approved drug against Ebola. In contrast, dexamethasone is a corticosteroid used to treat endocrine, rheumatic, collagen, dermatologic, allergic, ophthalmic, gastrointestinal, respiratory, hematologic, neoplastic, edematous conditions [6]. In [7–10] it was reported that baricitinib, used against RA with Janus kinase inhibitors, had been approved to be used on SARS-CoV-2 susceptible patients. Remdesivir act against SARS-CoV-2 by delaying chain cessation of nascent viral RNA [11–14]. [5] reported that baricitinib and remdesivir show improved clinical efficacy in the covid-19 patient when used together. Given the current situation of covid, remdesivir is mainly used against SARS-CoV-2 for clinical improvement and recovery of patients [15]. As reported in [5], baricitinib and remdesivir showed superior inhibitory effects than remdesivir alone in recovery rate among Covid-19 patients. Dexamethasone is a glucocorticoid receptor agonist that also acts as an immunosuppressive agent. [16, 17] reported that dexamethasone controls the effect of SARS-CoV-2 by combating cytokine storm in patients. Following a trial in the UK, dexamethasone is recommended for mechanically ventilated patients with COVID-19 or those who require supplemental oxygen [18].

There are several approaches for drug repurposing in COVID-19 that havealready been applied. Protein network and pathway approaches were used to derive the repurposed drug [19, 20]. In [21], a virtual docking screening and molecular dynamics simulation study of approved drugs and drug candidates in clinical pathology wereperformed to repurpose inhibitory drugs. Carfilzomib, Eravacycline, Valrubicin, Lopinavir, and Elbasvir were reported as the best protease inhibitor of SARS-CoV-2. In [22], methods similar to our approaches were used to discover talampicillin and lurasidone and two drug-like compounds ZINC000000702323 and ZINC000012481889, as potential anti-SARS-CoV-2 drugs.

To repurpose the three reference drugs for SARS-CoV-2, we have used their chemical-chemical and chemical-protein interactions based on the few publicly available SARS-CoV-2 target genes as described in section 0.2 0.4, and also built a drug similarity model. To constructthe drug similarity model [23], we have applied unsupervised machine learning approaches using the pharmacokinetics and drug likeliness of candidate compounds as features. The combination of techniques such as t-SNE, HAC, and OPTICs, which were used in developing the drug similarity model, render our presented approach unique.

**Fig 1:**
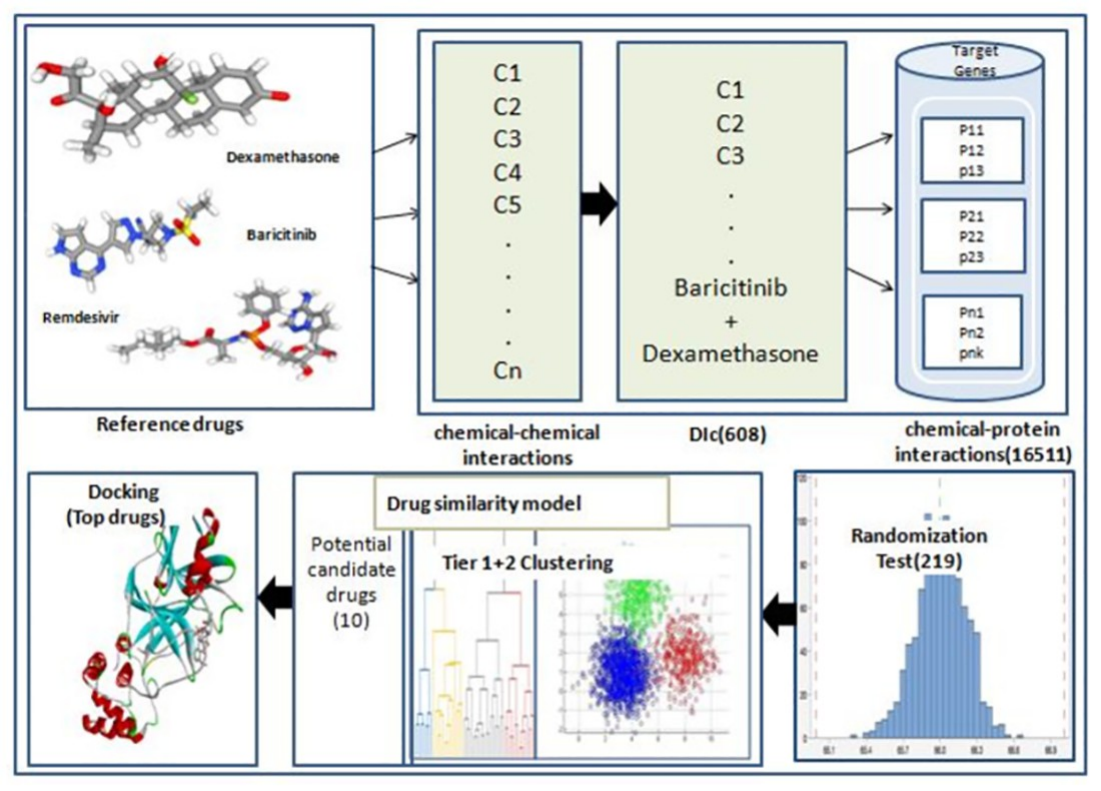
Stepwise workflow of repurposing three reference drugs as anti-COVID-19 drugs.

We show a graphical overview of the proposed workflow in Fig 1.

## Materials and methods

**Fig 2:**
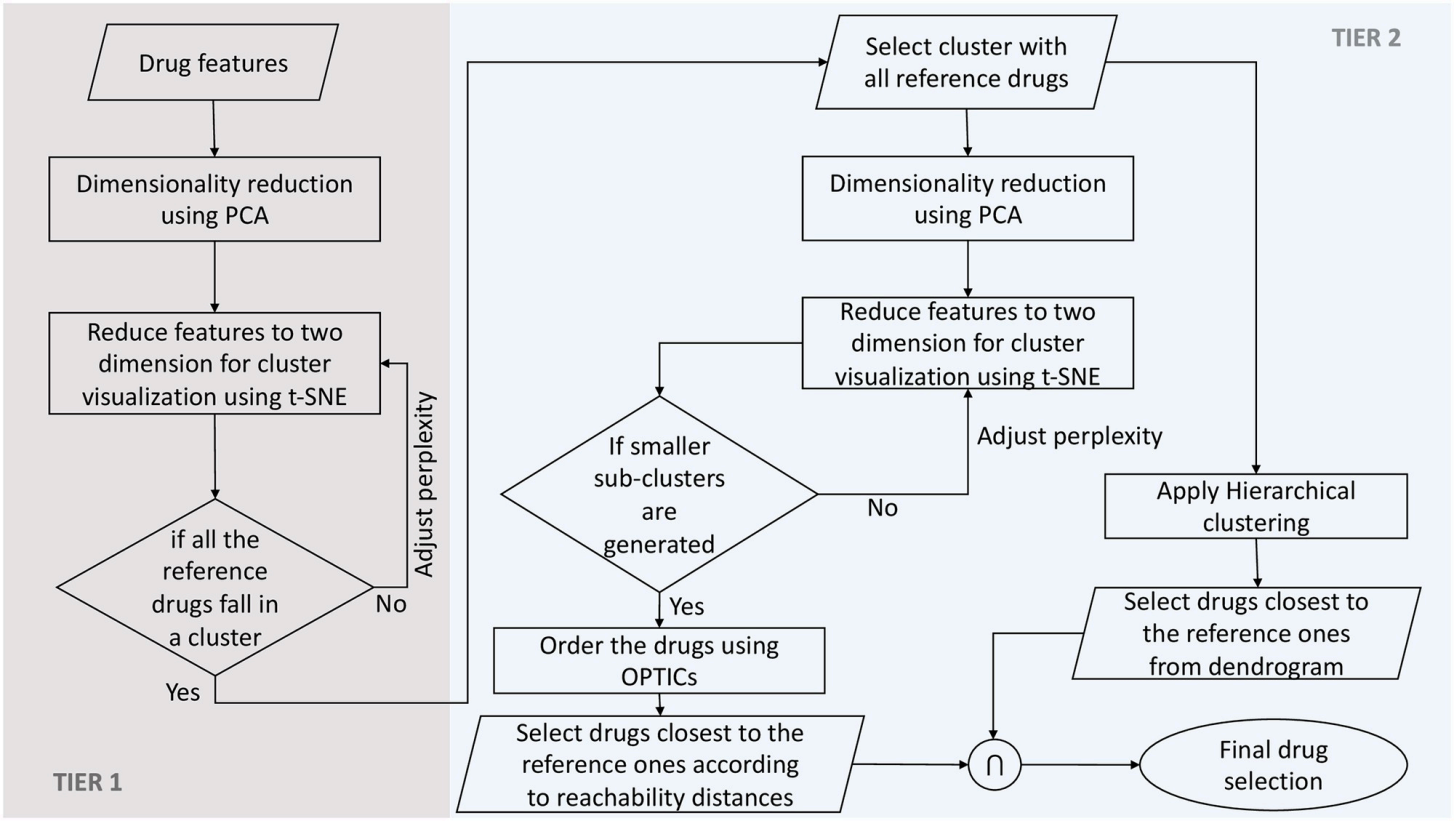
Flowchart of Two-Tier clustering approach.

Our study applied a unique drug similarity model to repurpose available drugs for SARS-CoV-2 by using three reference drugs based on their chemical-chemical and chemical-protein interactions. In search of new drug indications, we have used a two-tiered clustering approach, which is summarised in Fig 2. We have also applied this study pipeline toalternative drug sets, and our findings are given in S1 File.

### 0.1 Reference drugs

**Fig 3:**
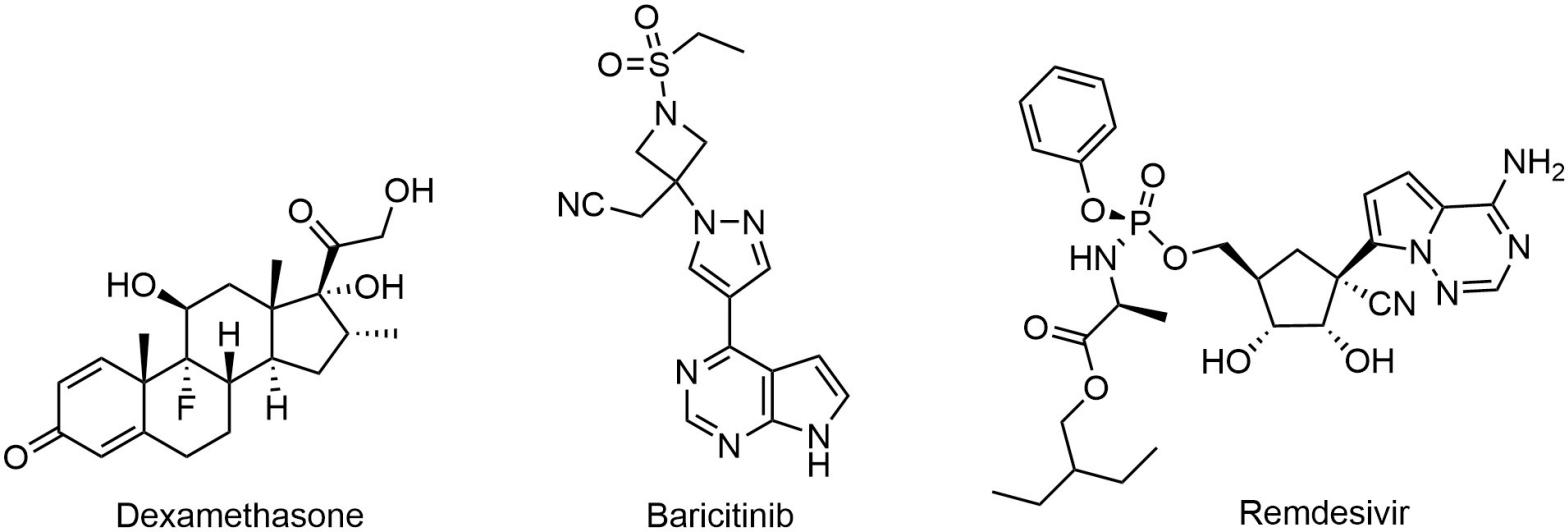
Chemical structure of considered reference drugs: (a) Dexamethasone (b) Baricitinib (c) Remdesivir.

Dexamethasone, baricitinib, and remdesivir are three drugs used as reference anti-SARS-CoV-2 drugs to find other potential drugs for SARS-CoV-2. Their chemical structures are shown in Fig 3.

### 0.2 Target genes

Target genes consisted 195 unique human target genes were retrieved from [24, 25]. [24] resulted in 163 target genes that were extracted by application of a new tensor decomposition based unsupervised feature extraction method on multiple gene expression profiles of lung cancer cell lines infected by SARS-CoV-2. Thirty-four gene sets were taken from [25] in which they first analysed the genome sequence of SARS-CoV-2 and identified sars as the most genomically similar disease, followed by MERS and other human Coronavirus diseases. We used those genes as an anti-SARS-CoV-2 target in our study (refer to S1 Table).

### 0.3 Tools and databases

Reference IDs and structures of the three reference drugs were downloaded from the PubChem database [26]. PubChem, Drugbank [6], and Drugcentral [27] have been used to search pharmacological properties of filtered candidate repurposed drug compounds. Chemical-chemical and chemical-protein interactions of reference drugs and their interactive compounds were downloaded from STITCH-2 database [28]. SwissADME(SIB) [29] was used for molecular descriptor calculations while performing the clustering. Autodock 4 was used for docking purposes and Discovery Studio (DS) 2.5(Accelrys Software Inc., San Diego, U.S.A.) for the docked structure analysis.

### 0.4 Chemical-Chemical and chemical-Protein interaction

It is crucial to identify and characterise the relationship between candidate anti-SARS-CoV-2 drugs and their target genes. We have used chemical-chemical and chemical-protein interactions for this purpose. The reason behind using interactive compounds is that such compounds often share similar functions [28, 30]. They may have similar structures, activities, or reactions, thus hinting that they could share the same pathways. This fact hypothesised that interactive compounds of the reference anti-SARS-CoV-2 drugs might also be involved in the same pathways related to SARS-CoV-2. Hence, it is essential to use them to discover new indications against SARS-CoV-2 infection.

In detail, first, the chemical-chemical and chemical-protein interaction data were downloaded from STITCH-2 [31–34]. The chemical-chemical and chemical-protein interaction data consists of different scores for each pair of compounds (similarity, experimental, database, text mining, and combined score). The Combined_score_ was used to define interactive compounds (for chemical−chemical and chemical-protein interactions): two compounds were considered interactive if their Combined_score_ was larger than zero [35]. The steps followed to derive candidate anti-SARS-CoV-2 drugs are as such:

First, we created a DIc file, a compound list consisting of two reference drugs and their chemical interactions. Since remdesivir had no interactions reported in the STITCH database, it could not be used for the initial interaction filtration. For all compounds in DIc, we extracted protein interactions where the proteins were the target genes of Saras-cov2. Baricitib had no such interactions that overlapped with the target genes. As baricitinib has a positive effect against SARS-CoV-2, all the proteins interactions were kept downstream, considering them as targets. Deterministic values for each compound were calculated by taking the mean of Combined_score_ of all interacting proteins. For example, if compound 1 (C1) is interacting with four proteins (P1, P2, P3, P4), then its deterministic value will be the mean of Combined_score_ of C1-P1 C1-P2, C1-P3 and C1-P4. We selected those compounds with a determination value greater than 0 as candidate drugs because the higher determination values can be interpreted as a strong association with the target genes of SARS-CoV-2; A drug compound with a determination value of 0 is unlikely to have the ability to act on SARS-CoV-2 pharmacologically.

### 0.5 Randomisation test

We performed a randomisation test to minimise false discoveries obtained in section 0.4. The adapted approach is similar to [35]. Drugs were selected using the cutoff of 0.05.

### 0.6 Drug similarity model: A Two-Tier clustering approach

To remove left out false discoveries and obtain the most similar and effective candidate compounds in terms of mechanism of action, we developed a final drug similarity model. This model is based on two-tiered clustering, as described below. The machine learning library sci-kit-learn [36] written in Python was used for this analysis. Subsequent clustering aimed to group the drugs that had similar features to the reference drugs. Fig 2 shows the flow diagram of the proposed approach. Tier-1 aims to identify outlier compounds, whereas the end product of Tier-2 gives us the drugs that are similar to the reference ones by running two independent clustering techniques in parallel. The unsupervised clustering method as applied herein to develop a drug similarity model is straightforward and can be easily applied and expanded in other research fields.

#### 0.6.1 Features selection for clustering

To input a drug similarity model, we used physicochemical, pharmacokinetic, and drug-like features of retained compounds. All the features were computed using the open-source online tool SwissADME [29, 37].

Pharmacokinetics provides a criterium to mathematically assess the effects of drugs in the body [38]. Drug-likeness is the parameter that qualitatively evaluates the bioavailability of drugs [39]. This parameter is calculated in terms of five rule-based methods: Lipinski (Pfizer) filter, Ghose (Amgen), Veber (GSK), Egan (Pharmacia), and Muegge (Bayer) [29, 40]. In addition, other calculated descriptors are described in [29].

Pharmacokinetics is a field that describes the processes which involve drug absorption, distribution, metabolism, and excretion (ADME), and such parameters can be used for the clustering of drug compounds. These parameters also play an essential role in ensuring that the drug achieves ultimate efficacy in fulfilling the medical need. It has also been observed that the probabilities of pharmacokinetics-related failure of a drug in the initial clinical trials are drastically reduced by prior estimation of ADME parameters in the drug discovery process [41, 42].

In [35], the clustering was performed on nine features (Similarity; Experimental; Database; Text mining; Combined score derived from chemical-chemical interactions dataset and other four features: Experimental; Database; Text mining; Combined_score derived from chemical-protein interactions data. However, these features were already used indirectly in earlier steps to predict chemical to chemical and chemical to protein interactions. Hence, using them in clustering might not give a clear and accurate drug discovery result. We have adapted physicochemical, pharmacokinetic, and drug-like features, which are non-repetitive and are essential parameters for drug discovery (listed in S2 Table). The selection of compounds based on these parameters increases the chances of drug success in passing the clinical trials.

#### 0.6.2 Tier-1 analysis

In Tier-1, Principal Component Analysis (PCA) [43] and t-Distributed Stochastic Neighbor Embedding (t-SNE) [44] were used for reducing the dimensionality for better visualisation of the well-separated drug groups. For better clustering of compounds, we followed a similar approach as in [45]. However, PCA is designed for an orthogonal transformation, resulting in linear combinations of features (PCs) to replace possibly correlated original features. In contrast, t-SNE is a non-linear stochastic algorithm which maps multi-dimensional data to a low dimension suitable for human reasoning while effectively grouping local data points close to each other. At first, PCA was performed, and its output PCs were fed to the t-SNE algorithm to deal with noise and achieve better isolation of clumps. The number of PCs was driven by explaining at least 99.5% of the total variance. This led to choosing initial 200 PCs for further analysis. The hyperparameter for the t-SNE algorithm is the “perplexity,” related to the number of nearest neighbours. The perplexity parameter varied from 5 to 500, and the output of the t-SNE manifold was observed 50 times for each iteration. For perplexity equals 170, the data distribution in two-dimensional space showed a consistent pattern where all three reference drugs fell into a single clump. Therefore, 122 out of 203 drug compounds within this clump were selected for the Tier-2 analysis.

#### 0.6.3 Tier-2 analysis

We have used two simultaneous yet independent clustering processes in Tier-2 on the filtered data set from Tier-1 analysis to achieve more reliable predictions. In one of the clustering branches, we used Hierarchical Agglomerative Clustering (HAC) [46], where each observation was considered as a singleton cluster in the beginning. Then sequentially, clusters were merged based on Ward’s minimum variance criterion [47].

The HAC method is sensitive to noisy data [48, 49], so we performed another density-based clustering method in parallel called OPTICS [50]. The hyperparameter in OPTICS is the minimum number of points (*MinPts*) to be considered a cluster member. It was kept at 5. The ordering was achieved by estimating the reachability distance between two points (*o*, *p*) defined as

$$\begin{array}{c} d\left(o,p\right)=\{\begin{array}{cc} \epsilon , & \text{if}\;\epsilon^{\prime }\leq \epsilon \\ \epsilon^{\prime }, & \text{otherwise} \end{array} \end{array}$$

where *ϵ* is the minimum Euclidean distance radius encircling at least *MinPts* points around a distinct point *p* called ‘core point’. *ϵ*′ is the euclidean distance between any point *o* and *p*.

Common drug compounds found in both the clustering method were chosen as the potential candidates foranti-SARS-CoV-2 drugs.

### 0.7 Molecular docking

The inhibiting potency of proposed drugs has been investigated by molecular docking against SARS-CoV-2 main protease 3CL or Chymotrypsin-like protease (PDB ID: 6LU7). The main protease performs a vital function in the viral maturation step by cleaving the non-structural proteins (Nsp). Nsp, which plays an essential role in viral replication, is digested at 11conservative sites [51]. Moreover, it mediates the assembly of replication-transcription machinery in the viral life cycle [52], and at the same time, it lacks closely related homologues in humans [53]. These reasons make MPro an ideal and mostly used antiviral target for docking against proposed drugs [54–56]. For this purpose, the development of specific inhibitors of the COVID-19 main protease can be of great importance in proposing the treatment regimen. From the drug similarity model, ten new drugs were obtained. Out of ten drugs, top drug candidates were filtered based on a threshold value. This threshold(487) was decided by the average deterministic value of reference drugs. Since the deterministic value was not available for remdesivir, the threshold value contained information from dexamethasone and baricitinib (561 and 413, respectively). Docking analysis was used to understand the working mechanism and validate the interaction of selected candidate drugs. To compare their inhibition potential, three reference drugs and the drugs that crossed the threshold value were docked with the same target.

**Target and ligand preparation**. Crystal structures of ligands were downloaded from the PubChem database [25], and of receptor SARS-CoV-2 main protease 3CL, Virus RdRp (PDBID: 7BV2) and glucocorticoid receptor (PDBID: 1M2Z) were taken from Protein Data Bank (PDB) (http://www.rcsb.org/pdb/). Chimera was used for ligand structure minimisation using AMBER ff14SB forcefield, and proteins were prepared and docked using the Autodock 4 tool [60]. Furthermore, water (HOH) was removed during the editing of native PDB files of the selected 3D structure of COVID-19proteins, i.e. main protease (PDB: 6LU7) and peptidase (PDB:2GTB). The hydrogen atoms, Kollman united charges, and the default solvation parameters were added to both proteins. Gasteiger charge is also assigned to the drug compounds. Grid dimensions were selected based on the binding pocket of ligand bound to the target protein, i.e. E3. Grid Centre dimensions were as such:X = -12.0443, y = 10.626, z = 68.664. Docking calculations were based on the Lamarckian Genetic Algorithm (LGA) [60]. One hundred runs of LGA were used to get the final execution of docking. Finally, obtained conformations of docked complexes were analysed for different interactions using Discovery Studio (DS) molecular visualisation software. Binding energy and the number of H-bonds were used to compare the effective binding of proposed drugs with the target protein. The highly negative binding energy and less inhibitory constant represent better binding efficiency hence considered a better drug. The data downloaded from the databases mentioned above are provided in S2 File.

## Results

### 0.8 Chemical-chemical and chemical-protein interaction study

Chemical-chemical and chemical-protein interaction search for three reference drugs as an initial step for a potential anti-SARS-COV-2 drug search. There was no chemical interaction available for remdesivir in the STITCH database. Hence we started the data mining for candidate drugs with dexamethasone and baricitinib and included remdesivir in the drug similarity model to get the final list of proposed anti-SARS-COV-2 drugs. DI_C_ contained 608 compounds, including two reference drugs(dexamethasone and baricitinib) and their interactive compounds (refer to S3 Table). These compounds and the 195 target genes(mentioned in section 0.2) were used to obtain a total of 16511 unique protein interactions for all compounds in DI_C_. The determination values for each compound were calculated by using the method described in Section 0.4. If one compound in DI_C_ was not involved in any chemical-protein interaction, its determination value was set to zero. Finally, 387 compounds with determination values greater than zero were obtained (S4 Table).

### 0.9 Randomization test

Based on the randomisation test, 218 Compounds fell below the threshold and were chosen to pass through feature extraction. Since we included remdesivir as another reference drug to be used in the drug similarity model hence, in total, 219 compounds were fed to SWISSADME. In total, 203 hits were found and were used for further downstream analysis.

### 0.10 Drug similarity model

**Fig 4:**
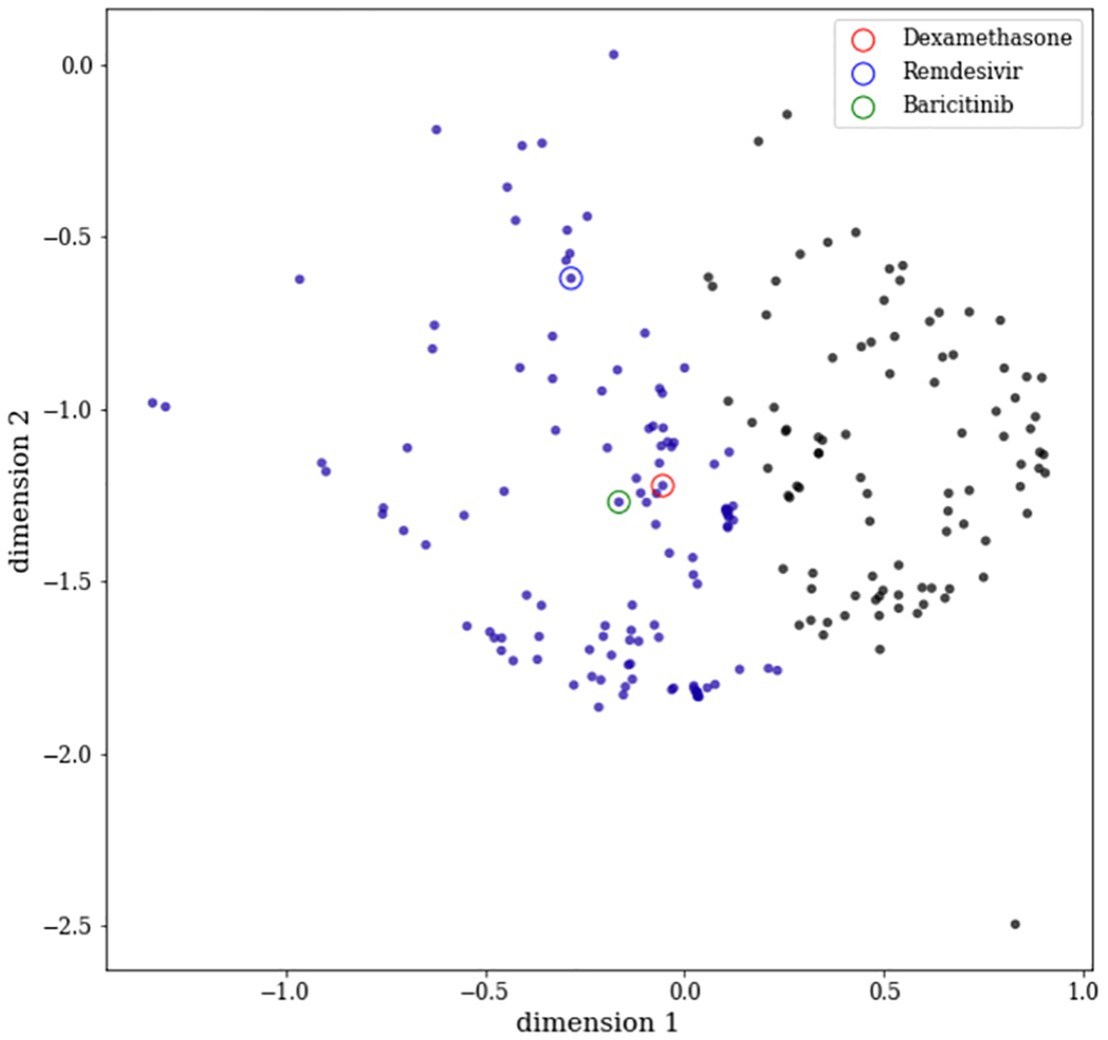
The output of t-SNE dimensionality reduction using a perplexity of 170 from 200 principal components. It is to be noted that the clump with purple colour code contains three reference drugs. The colours on each data point were obtained as a result of applying a simple K-means clustering assuming 2 clusters.

**Tier-1 clustering**. A total of 203 drug compounds, including the approved drugs dexamethasone, baricitinib, and remdesivir, were used to develop the drug similarity model. The drug group consisting of three approved drugs was identified using the combination of PCA and t-SNE (see Fig 4) as described in Section 0.6.2. We filtered out the 81 drugs which did not show any association with the approved drugs, and the remaining 122 drugs were considered for the next level of clustering.

#### Tier-2 clustering

**Fig 5:**
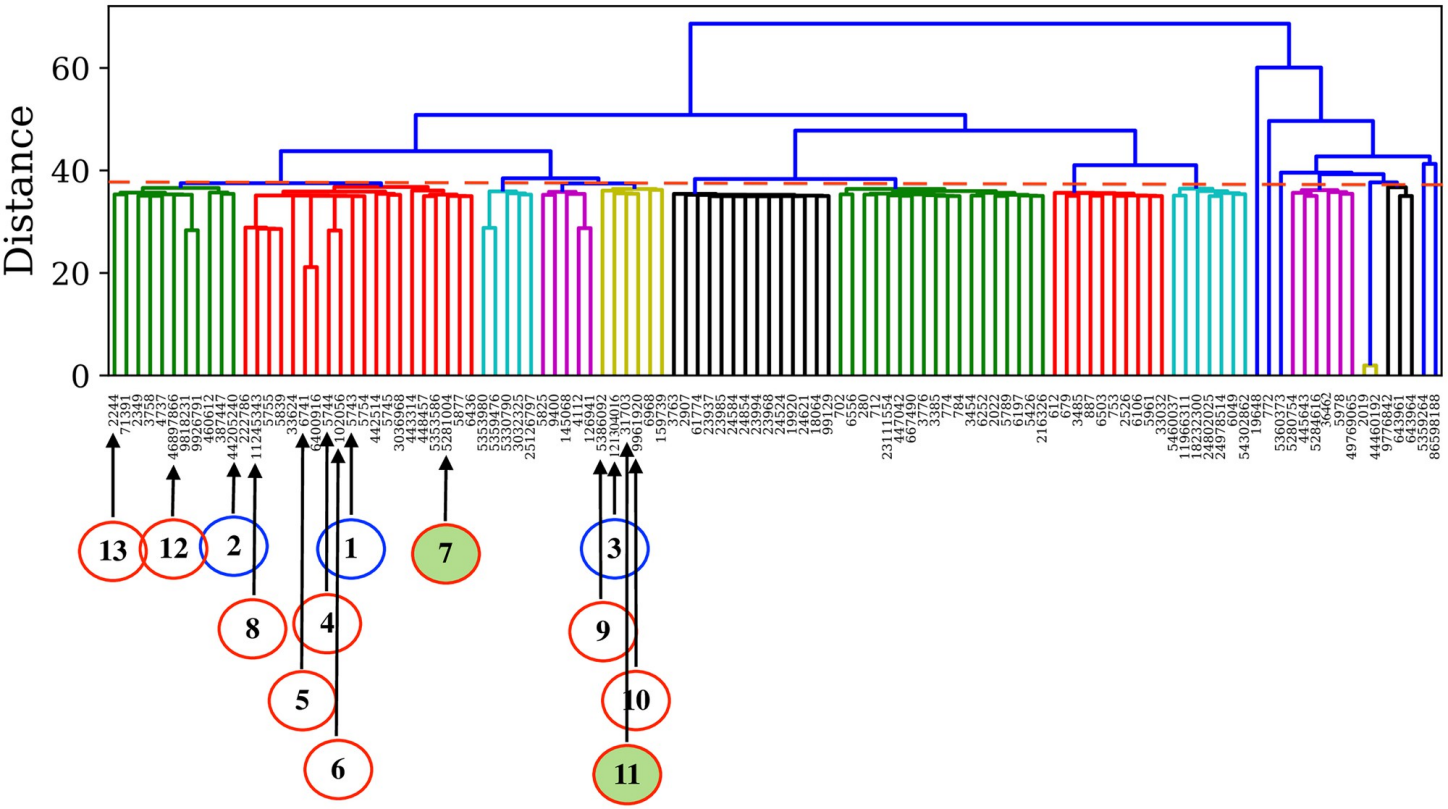
Dendrogram obtained from HAC showing three different clusters associated with the reference drugs. The distance (represented by a dashed red line) is chosen at 38 for the cluster formation. The reference drugs and the top 10 similar drugs quoted in Table 1 are indicated using blue and red circles, respectively, with connected arrows. The best two drug candidates are highlighted via filled red circles.

**Fig 6:**
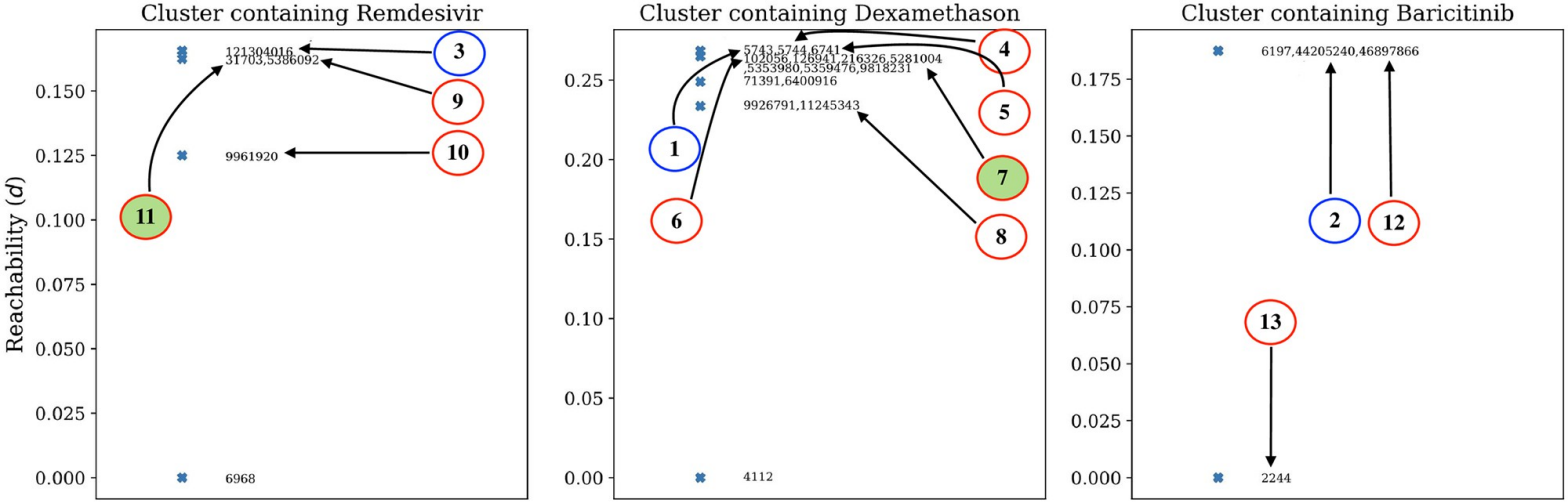
Reachability plot of three different clusters associated with the reference drugs obtained from OPTICS analysis. The occurrence of the reference drugs, similar drugs and the best two candidate drugs in the reachability plot are highlighted in a similar manner as that of Fig 5.

The two independent and parallel clustering approaches, applied to the 122 selected drugs, could further demonstrate the high similarity of a few drug compounds with the three reference drugs. From HAC implementation, we identified 37 such drug compounds. The hierarchical relationships between compounds are shown in the form of a dendrogram in Fig 5. OPTICS led to the identification of 24 such compounds. The reachability distance of associated cluster members with the reference compounds is shown in Fig 6. Thus, We could find ten compounds common in HAC and OPTICS clustering predictions. Details of these findings are summarised in Table 1.

**Table 1:** Predicted compounds for the treatment of SARS-CoV-2 related symptoms.

Sl No.	Compund ID	Name	Pubchem Reference	Determination Value	Clinical experience in COVID-19
1	5743	**Dexamethasone**	https://pubchem.ncbi.nlm.nih.gov/compound/5743	413	[17]
2	44205240	**Baricitinib**	https://pubchem.ncbi.nlm.nih.gov/compound/44205240	561	[5]
3	121304016	**Remdesivir**	https://pubchem.ncbi.nlm.nih.gov/compound/121304016	NA	[5]
4	5744	Hydrocortisone acetate	https://pubchem.ncbi.nlm.nih.gov/compound/5744	266	[57]
5	6741	Methyprednisolone(Medrone)	https://pubchem.ncbi.nlm.nih.gov/compound/6741	339	[58]
6	102056	1250–97–1(EP-Hydrocortisone Acetate)	https://pubchem.ncbi.nlm.nih.gov/compound/102056	249	[59]
7	5281004	**Buedesonide**(pulmicort)	https://pubchem.ncbi.nlm.nih.gov/compound/5281004	555	[60, 61]
8	11245343	Prednisolone hydrate	https://pubchem.ncbi.nlm.nih.gov/compound/11245343	287	[59]
9	5386092	Fungizone	https://pubchem.ncbi.nlm.nih.gov/compound/5386092	199	[62]
10	9961920	Ciprodex	https://pubchem.ncbi.nlm.nih.gov/compound/9961920	400	NA
11	31703	**Doxorubicin**	https://pubchem.ncbi.nlm.nih.gov/compound/31703	488	[63]
12	46897866	Olanzapine(O15)	https://pubchem.ncbi.nlm.nih.gov/compound/46897866	342	NA
13	2244	Aspirin	https://pubchem.ncbi.nlm.nih.gov/compound/2244	479	[64]

Reference drugs and the names of three drugs having the highest determination value are highlighted in bold letters. The bold shows reference drugs and the top two drugs selected based on the determination threshold.

### 0.11 Molecuclar docking

**Fig 7:**
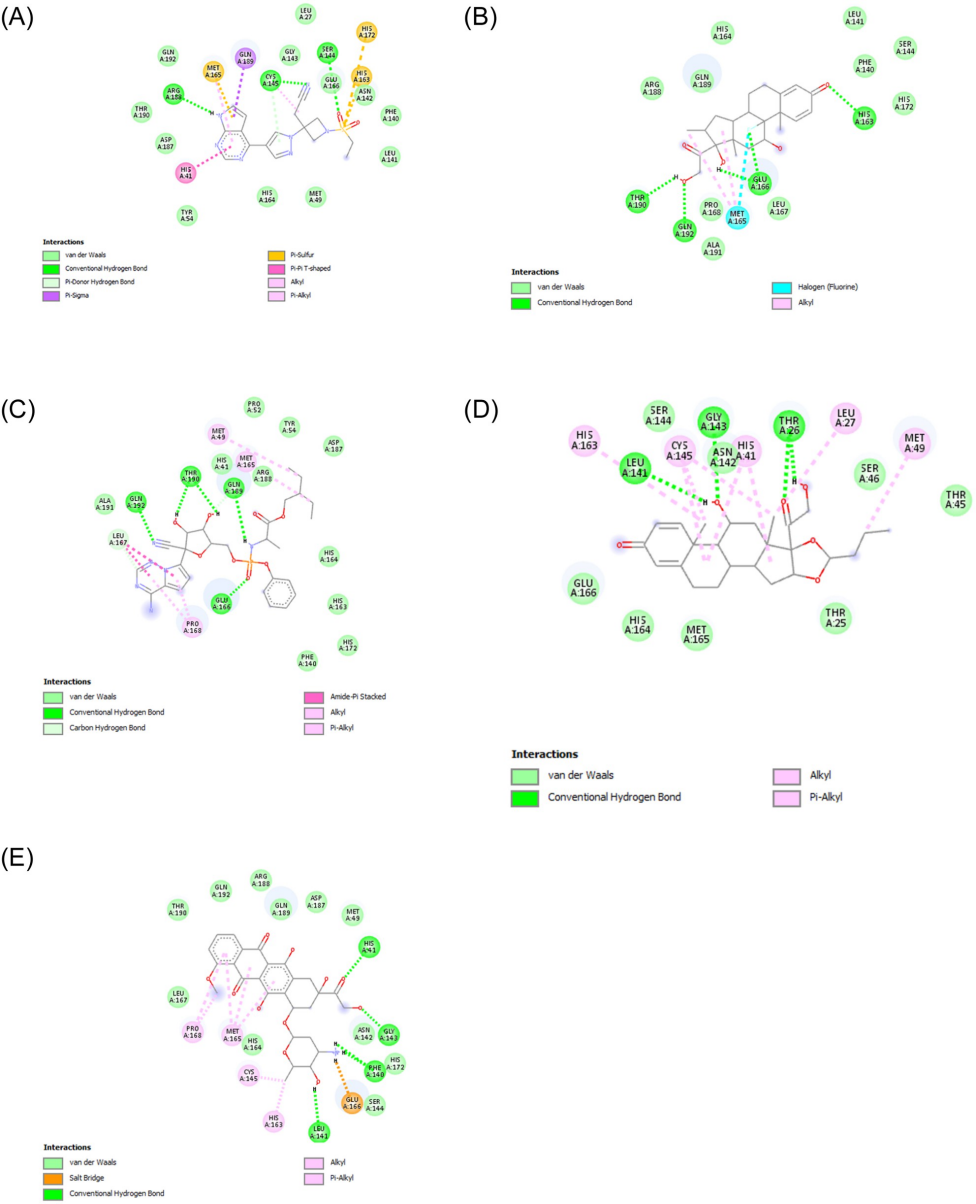
2D interactions of candidate drugs with target protein. **A**. 2D bond-graph of baricitinib binding with target. **B**. 2D bond-graph of dexamethasone binding with target. **C**. 2D bond-graph of remdesivir binding with target. **D**. 2D bond-graph of budesonide binding with target. **E**. 2D bond-graph of doxyrubicin binding with target.

The average deterministic value of reference drugs (see section 0.7)served as an additional threshold (487) to further prioritise drugs for molecular docking. Based on the deterministic values of each of the ten drugs obtained in Section 0.4, only budesonide and doxorubicin (respective deterministic values of 555 and 488) passed this new threshold(see also Table 1). Hence, these two drugs were chosen for docking analysis and three reference drugs. The binding of drugs was compared based on two parameters: binding energy and inhibition constant. Drugs having the lowest binding energy and inhibition constant were considered the best drugs Table 2. Doxorubicin had comparable binding energy(-8.80 Kcal/mol) and the least inhibitory constant(459.78 nM) against target protein mentioned in Table 2. Binding energy and inhibitory constant of budesonide, dexamethasone, baricitinib and remdesivir with MPro were found to be: -6.05(kcal/mol) and 8.83(*μ*M), -8.08(kcal/mol) and 1.2(*μ*M), -8.85(kcal/mol) and 326.41(nM), -8.96 (kcal/mol) and 272.24(*μ*M) respectively. Bonds formed in docking of SARS-Cov-2 target protein and proposed drugs are shown in Fig 7A to 7E. 2D graphs plotted with the help of Discovery Studio Visualizer 2017 R2. Dashed lines and different colours represent the various interactions and the interaction type, respectively. Residues of target protein(MPro) involved in hydrogen bonding with doxorubicin: His41, GLY143, PHE140, LEU141. MPro formed HBond with Buedesonide with: LEU141, THR26(2), GLY143, with baricitinib:ARG188, SER144, CYS145, GLU166, with remdesivir:GLN192, GLN189, THR190(2), GLU166 and with dexamethasone:His163, GLU166, GLN192, THR190. The structures of candidate drugs and their docked complexes with the target are shown in S4 Table.

**Table 2:** Binding energy and drug inhibition constant for docking of drugs with SARS-CoV-2 main protease.

S.No	Drug	Binding Energy(Kcal/Mol)	Inhibitory constant	NO. of H-Bonds	Amino acid of target protein involved in H-bonding
1	Baricitinib	-8.85	326.41(nM)	4	ARG188,CYS145,SER144,GLU166
2	Dexamethasone	-8.08	1.2(uM)	4	His163,GLU166(2), GLN192,THR190
3	Remdesivir	-8.96	272.24(nM)	5	GLN192,THR190(2),GLN189,GLU166
4	Doxorubycin	-8.80	459.78(nM)	4	4HIS41,GLY143,PHE140,LEU141
5	Budesonide	-6.9	8.83(uM)	4	4LEU141,THR26(2),GLY143

## Discussion

Based on FDA approved drugs such as dexamethasone, baricitinib and remdesivir to treat patients infected with SARS-CoV-2, we have developed a drug similarity model to find other potential drug compounds for the treatment ofSARS-CoV-2 virus. Our study in section 0.5 resulted in 203 compounds, which had high determination values. Therefore, the 203 compounds acted as the initial sample for our analysis. Fifty features were selected based on molecular parameters of drug likeliness, physicochemical properties, and ADME of the filtered drug compounds mentioned in S2 Table. We have implemented unsupervised clustering techniques such as t-SNE, HAC, OPTICS (see Section 0.6.2, 0.6.3) to identify ten new potential drugs sharing similar properties with the reference drugs- dexamethasone, baricitinib and remdesivir. Other significant parameters, such as toxicity and ionisation properties, could have been used to populate this feature vector by using special software such as ADMETPredictor, ADMET-SAR and ProTox, Schrodinger(QuickPro), which was beyond the scope of this paper. We followed a sequential yet efficient approach thatfilters out the unrelated compounds with the reference drugs. First, we used the stochastic technique, namely t-SNE, to visually inspect and remove the dissimilar compounds in the first tier. Then, we used HAC and OPTICS in parallel to segregate the most promising drugs. These two independent clustering techniques are simple in their implementation, allowing better comprehension and control over each processing step. Our drug similarity model highlighted ten drugs from three reference drugs, as shown in Table 1, that can potentially act against the SARS-CoV-2 virus. A literature survey of all ten drugs was performed to gather sufficient evidence supporting their potential inhibitory role against the SARS-CoV-2 virus, as shown in Table 1. Pubchem [26] was used to search for previously reported drugs, the mechanism of action and other medicinal properties. We used the determination value to compare the inhibitory activity of the final ten drugs against SARS-CoV-2 targets under the dogma that the higher the determination value, the higher the association of drugs with SARS-CoV-2 targets. These values are a good measure to compare the efficiency of drugs to be successful in later clinical phases. Out of ten candidate drugs in our result, the determination values of budesonide: CID5281004 and doxorubicin(CID31703) surpassed the chosen cutoff value, indicating that these two drugs have comparatively higher potential to act as an anti-SARS-CoV-2 drug than the rest of the other eight drugs. Further validation of drugs using docking showed that doxorubicin could also inhibit this deadly virus by effectively interacting with its spike protein(main protease). Doxorubicin has antiviral activity and can decrease viral load and reduce the risk of vascular and renal complications of COVID-19 by increasing the cellular concentration of methylglyoxal MG in human host tissues [65]. Other candidate drugs could potentially demonstrate a similar inhibitory pattern when used in combination. Another interesting observation relating to pathways of drugs [66] lies within the fact that maximum(seven) of the ten reported drugs have the same target pathways, i.e., Neuroactive ligand-receptor interaction(hsa04080). Although these *in-silico* validations give positive indications towards the discovery of anti-SARS-CoV-2 drugs, further validation of the newly discovered drugs needs to be done. In addition, molecular dynamics studies can be performed to check their interactions with SARS-CoV-2 targets in humans and gather additional supporting pieces of evidence for our present results.

## Conclusion

This study proposed a unique method to discover potential candidate drugs for the SARS-CoV-2 virus. A list of small-molecule drugs was reported by investigating chemical-chemical and chemical-protein interactions followed by two-tier unsupervised clustering. These drugs may have the capability to treat SARS-CoV-2 infection. The potential of these candidate drugs was supported by an in-depth analysis of knowledge around the ten shortlisted compounds. Furthermore, interaction mechanisms of two filtered top drugs were tested and validated through docking studies. We hope that our approach may serve as an effective and reproducible aid for Computational driven drug repurposing.

## Supporting information

S1 File Study on alternative drug set. (PDF)

S2 File Structures of all the ligands and main target for SARS-CoV-2 virus. (DOCX)

S1 Table List of Target genes for SARS-CoV-2. (DOCX)

S2 Table Molecular features used for clustering. (DOCX)

S3 Table DIC containing Pubchem IDs of three reference drugs and all their chemical interactions. (TXT)

S4 Table Compounds with their determination value. (TXT)
