# Supplementary material for: Discovery of new drug indications for COVID-19: A drug repurposing approach
Source: PLoS One. 2022 May 24;17(5):e0267095. doi: 10.1371/journal.pone.0267095 (PMC9129022; doi:10.1371/journal.pone.0267095)
Supplement: S1 File — (PDF) [file pone.0267095.s001.pdf]

Study on alternative Drug set

1 Results based on analysis of Hydroxychloroquine(HCQ) and Chloroquine(CQ)

The proposed pipeline was applied to find out repurposed drugs for anticovid-19 using Hdroxychloroquine and Chloroquine along with Dexamethasone. Chemical-chemical and chemical-protein Interaction study based on HCQ and CQ resulted in DIC containing 888 compounds with determination values greater than zero.

1.1 Drug Similarity Model

Tier-1 Clustering:

A drug similarity model was built using a total of 774 drug compounds, with the three approved drugs cq, hcq, and dexamethasone. The drug group was identified using the combination of PCA and t-SNE fig 1. 446 drugs were filtered out which did not show any association with the approved drugs. In the end, 328 drugs remained for the next level of clustering.

Tier-2 Clustering :

328 selected drugsn were passed through tier-2 clustering. From HAC, we could identify 24 such drug compounds(refer to fig 2), whereas OPTICS provided us with 17 such compounds as shown in fig 3. We could find 16 compounds common in both HAC and OPTICS clustering predictions. The details of these findings are summarized in Table I.

Compund ID	Name	MOA	Determination Value	Clinical experience in COVID-19
4917[‡]	Prochlorperazine	antipsychotic	192	[1]
5566[‡]	Trifluoroperazine	antipsychotic and an antiemetic	334	[2]
**3652**[‡]	Hydroxychloroquine	antimalarial	250	[3]
3696[‡]	Imipramine	antidepressant	383	[3]
2995	Desipramine	antidepressant	400	[3]
444810	(R)-Chloroquine	Enantiomer of Chloroquine	385	[4, 5]
**2719**[‡]	Chloroquine	antimalarial	290	[3]
**3386**	**Fluoxetin**	antidepressant	510	[6]
2165	Amodiaquine	anti-inflammatory	355	[7]
65028[‡]	Oseltamivir	antiviral neuraminidase inhibitor	183	[8]
4027	CA-074 Methyl Ester	neuroprotective, anti-cancer, and anti-inflamatory effects	218	[9]
65663	Aloxistatin	cysteine protease inhibitor, anticoagulant	240	[10]
6087[‡]	Meticillin	anti Gram-positive bacteria	248	[11]
**33624**[‡]	**Timolol**	nonselective beta-adrenergic antagonist	647	[12, 13]
5839	Aldosterone	NA	380	[14]
20469	Beclomethasone	glucocorticoid receptor	419	[15]
20055008	Prednylidene	experimental systemic glucocorticoid	166	[16]
5744[‡]	Hydrocortisone Acetate	anti-inflammatory	233	[16]
102056[‡]	Epihydrocortisone 21-acetate	anti-inflammatory	198	[16]
**5743**[‡]	**Dexamethasone**	glucocorticoid receptor, anti-inflammatory	555	[17]
9782[‡]	Betamethasone	immunosuppressive and antiinflammatory	150	[16]
5865[‡]	Prednisone	anti-inflammatory glucocorticoid	296	[16]
5754[‡]	Hydrocortisone	anti-inflammatory	479	[16]
6741[‡]	Methylprednisolone	anti-inflammatory	432	[16]
6400916[‡]	SCHEMBL5085	NA	281	[16]
5755[‡]	Prednisolone	glucocorticoid receptor,anti-inflammatory	372	[16]
11245343[‡]	Prednisolone hydrate	glucocorticoid receptor,anti-inflammatory	325	[16]
222786[†]	Cortisone	anti-inflammatory	294	[16]

Table 1: Predicted compounds for COVID-19 based on HCQ and CQ
Reference drugs and the names of three drugs having highest determination value are highlighted in bold letters. [‡] The drug compounds found in both HAC and OPTICS methods, [†] the drug compounds only found from OPTICS method and unlabeled ones are only found from HAC analysis.

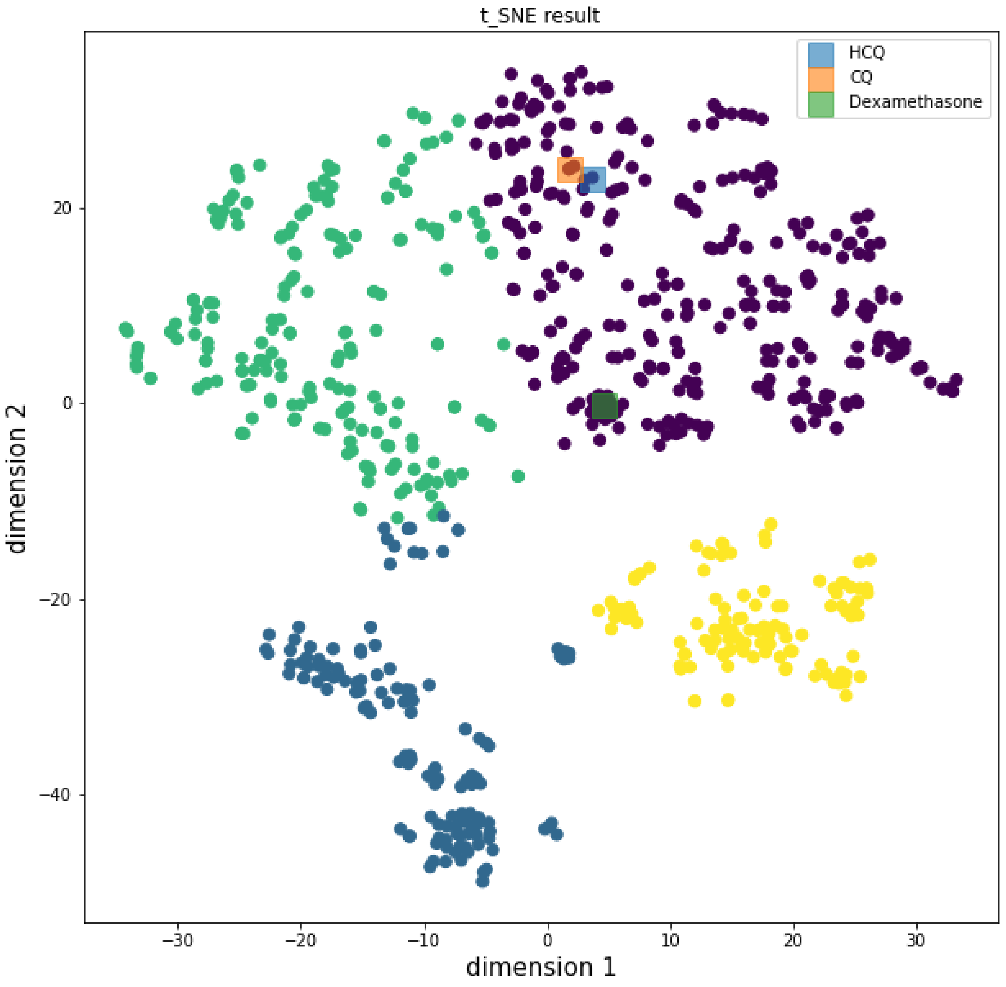

Figure 1: Output of t-SNE dimensionality reduction using a perplexity of 30 from 40 principal components. It is to be noted that the clumps in brown color contains all three reference drugs. The colors on each data point is a result of applying a simple K-means clustering assuming 4 number of clusters.

2 Discussion

The drug similarity model in our results highlighted 29 drugs based on three reference drugs (Table 1) that can potentially act against novel corona virus. Determination value is used to compare the inhibitory activity of final 29 drugs against COVID-19 targets. In recent clinical trials, Dexamethasone has performed much better than HCQ and CQ and reported to reduce deaths by one-third in mechanically ventilated COVID-19 patients, and by one-fifth in patients receiving oxygen only[18]. Higher determination value of Dexamethasone than HCQ and CQ provides support for this observation. Out of 29 drugs,determination values of Timolol(647) has surpassed that value of Dexamethasone. This shows that Tomilol has the highest potential to perform best among all in the list when used in clinical trials against novel corona. Fluoxetin having determination value 510 near to Dexamethasone possess the similar inhibitory pattern. Drugs having detrmination value lower than 250 for example- compounds with CID 4917,65028,4027,65663,6087,5744,102056,9782,6335487 show less association with

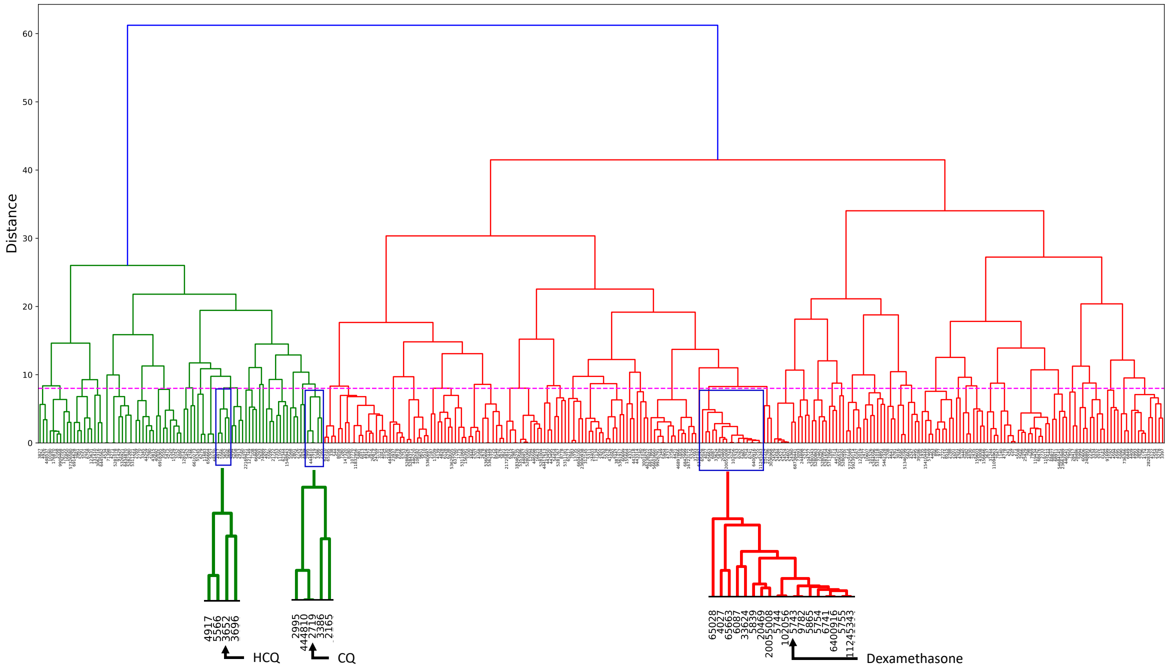

Figure 2: Dendrogram obtained from HAC showing compounds within a same cluster as that of the reference drugs. The distance (represented by dashed magenta line) is chosen in such a way that there remains at least three compounds associated with each reference drug.

corona. Literature survey of all drugs was done to gather evidences for their inhibitory potential against novel corona virus. Drugbank [19]) and Drugcentral [20] and Stanford Coronavirus Antiviral research database (https://covdb.stanford.edu) were used to search the mechanism of action(MOA) and other details of drugs. Out of 29 drugs, most of the drugs belong to class steroid and its derivative. Two are Benzothiazines and others are either carboxyl aid and its derivatives or Benzene derivatives. Only Timolol is the compound which belongs to Organonitrogen compounds. Majority of the drugs are anti-inflammatory agents. We verified our result with various literatutes mentioned in Table1. Few drug compounds from this list are already reported as labeled and off-labeled indications for COVID-19. This itself can be considered as one of the validation of our result. Others are newly discovered.

References

[1] Christina L Szperka, Jessica Ailani, Rebecca Barmherzig, Brad C Klein, Mia T Minen, Rashmi B Halker Singh, and Robert E Shapiro. Migraine care in the era of covid-19: Clinical pearls and plea to insurers. *Headache: The Journal of Head and Face Pain*, 60(5):833–842, 2020.

[2] Yiyue Ge, Tingzhong Tian, Sulin Huang, Fangping Wan, Jingxin Li, Shuya Li, Hui Yang, Lixiang Hong, Nian Wu, Enming Yuan, et al. A data-driven drug repositioning framework discovered a potential therapeutic agent targeting covid-19. *bioRxiv*, 2020.

[3] Oleg O Glebov. Understanding sars-cov-2 endocytosis for covid-19 drug repurposing. *The FEBS Journal*, 2020.

[4] Ilaria D'Acquarica and Israel Agranat. Chiral switches of chloroquine and hydroxychloroquine: potential drugs to treat covid-19. *Drug Discovery Today*, 2020.

[5] Guanguan Li, Jing Sun, Yingjun Li, Yongjie Shi, Jincun Zhao, Tony Y Zhang, and Xumu Zhang. Enantiomers of chloroquine and hydroxychloroquine exhibit different activities against sars-cov-2 in vitro, evidencing s-hydroxychloroquine as a potentially superior drug for covid-19. *bioRxiv*, 2020.

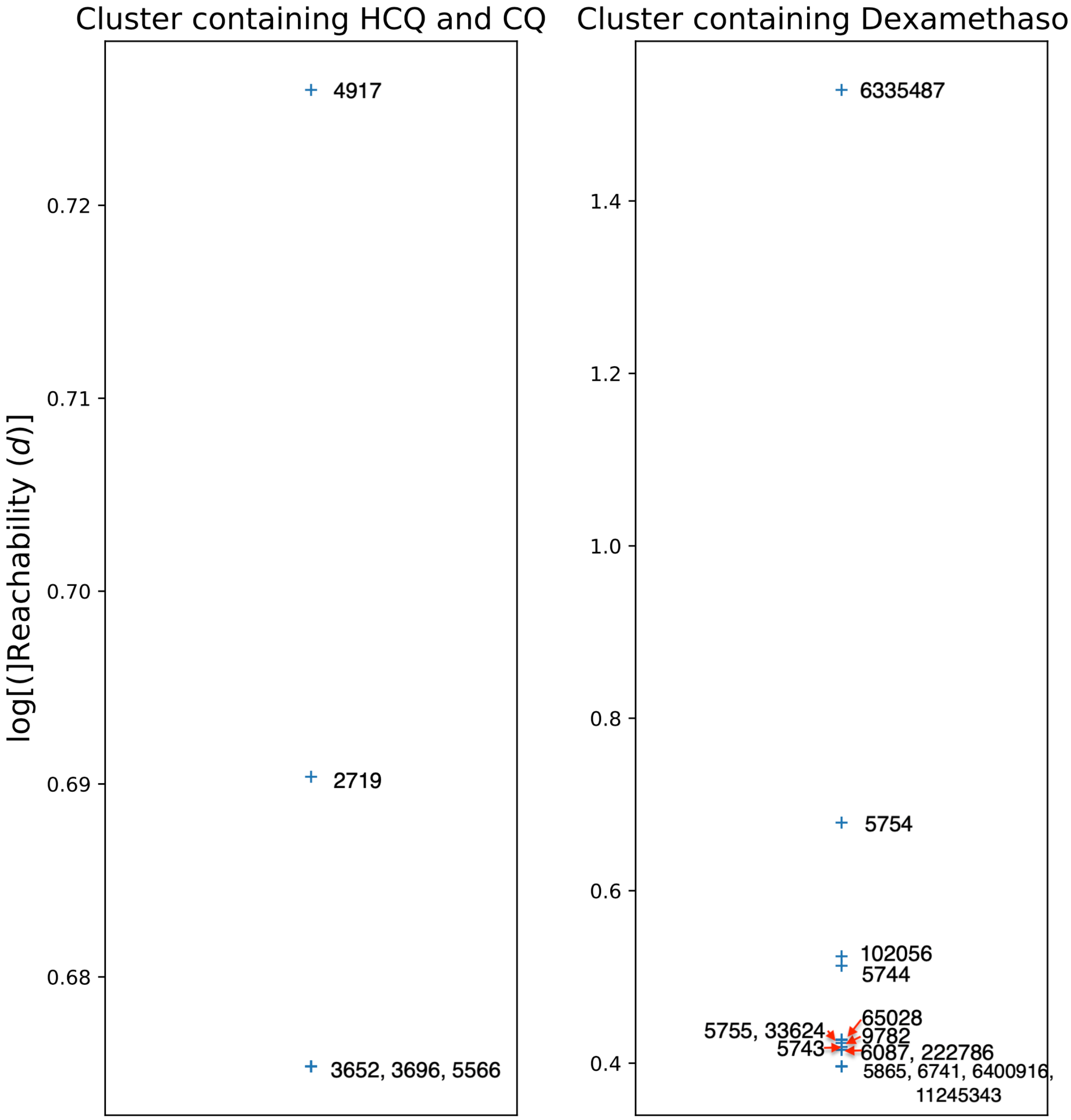

Figure 3: Reachability plot of different compounds obtained from OPTICS analysis.

[6] Melissa Zimniak, Luisa Kirschner, Helen Hilpert, Jürgen Seibel, and Jochen Bodem. The serotonin reuptake inhibitor fluoxetine inhibits sars-cov-2. *Biorxiv*, 2020.

[7] Longlong Si, Haiqing Bai, Melissa Rodas, Wuji Cao, Crystal Yur Oh, Amanda Jiang, Atiq Nurani, Danni Y Zhu, Girija Goyal, Sarah Gilpin, et al. Human organs-on-chips as tools for repurposing approved drugs as potential influenza and covid19 therapeutics in viral pandemics. *bioRxiv*, 2020.

[8] Nisha Muralidharan, R Sakthivel, D Velmurugan, and M Michael Gromiha. Computational studies of drug repurposing and synergism of lopinavir, oseltamivir and ritonavir binding with sars-cov-2 protease against covid-19. *Journal of Biomolecular Structure and Dynamics*, pages 1–6, 2020.

[9] Tianxiao Liu, Songyuan Luo, Peter Libby, and Guo-Ping Shi. Cathepsin l-selective inhibitors: A potentially promising treatment for covid-19 patients. *Pharmacology & Therapeutics*, page 107587, 2020.

[10] Xiuyuan Ou, Yan Liu, Xiaobo Lei, Pei Li, Dan Mi, Lili Ren, Li Guo, Ruixuan Guo, Ting Chen, Jiaxin Hu, et al. Characterization of spike glycoprotein of sars-cov-2 on virus entry and its immune cross-reactivity with sars-cov. *Nature communications*, 11(1):1–12, 2020.

[11] Ailan Huang, Xue Tang, Huimin Wu, Jun Zhang, Wanqi Wang, Zhiwei Wang, Li Song, Min-an Zhai, Lihui Zhao, Hailong Yang, et al. Virtual screening and molecular dynamics on blockage of key drug targets as treatment for covid-19 caused by sars-cov-2. 2020.

[12] Ayman Farag, Ping Wang, Mahmoud Ahmed, and Hesham Sadek. Identification of fda approved drugs targeting covid-19 virus by structure-based drug repositioning. *ChemRxiv, doi: doi*, 10, 2020.

[13] Khan Sharun, Ruchi Tiwari, Jaideep Dhama, and Kuldeep Dhama. Dexamethasone to combat cytokine storm in covid-19: Clinical trials and preliminary evidence. *International journal of surgery (London, England)*, 2020.

[14] Harald Murck. Symptomatic protective action of glycyrrhizin (licorice) in covid-19 infection? *Frontiers in Immunology*, 11:1239, 2020.

[15] Daisuke Miyazawa and Gen Kaneko. Clinical trials of inhaled beclomethasone and mometasone for covid-19 should be conducted. *Journal of Medical Virology*, 2020.

[16] Cinzia Solinas, Laura Perra, Marco Aiello, Edoardo Migliori, and Nicola Petrosillo. A critical evaluation of glucocorticoids in the treatment of severe covid-19. *Cytokine & growth factor reviews*, 2020.

[17] Raymond M Johnson and Joseph M Vinetz. Dexamethasone in the management of covid-19, 2020.

[18] Claudio Ortolani and Elide A Pastorello. Hydroxychloroquine and dexamethasone in covid-19: who won and who lost? *Clinical and Molecular Allergy*, 18(1):1–7, 2020.

[19] David S Wishart, Yannick D Feunang, An C Guo, Elvis J Lo, Ana Marcu, Jason R Grant, Tanvir Sajed, Daniel Johnson, Carin Li, Zinat Sayeeda, et al. Drugbank 5.0: a major update to the drugbank database for 2018. *Nucleic acids research*, 46(D1):D1074–D1082, 2018.

[20] Oleg Ursu, Jayme Holmes, Jeffrey Knockel, Cristian G Bologa, Jeremy J Yang, Stephen L Mathias, Stuart J Nelson, and Tudor I Oprea. Drugcentral: online drug compendium. *Nucleic acids research*, page gkw993, 2016.
