# Supplementary material for: Discovery of new drug indications for COVID-19: A drug repurposing approach
Source: PLoS One. 2022 May 24;17(5):e0267095. doi: 10.1371/journal.pone.0267095 (PMC9129022; doi:10.1371/journal.pone.0267095)
Supplement: S2 File — (DOCX) [file pone.0267095.s002.docx]

**Structures of ligands and docked complex of candidate drugs with target(MPro) compound ds**

| **S. NO.** | **Comp** | **CID** | **Ligand** | **Docked Structures** |
| --- | --- | --- | --- | --- |
| 1 | Budesonide | 5281004 | 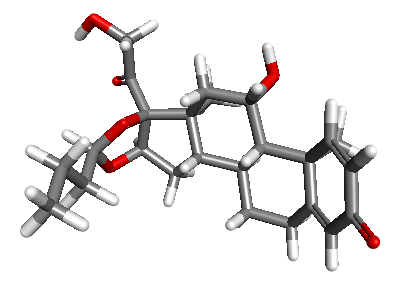 | 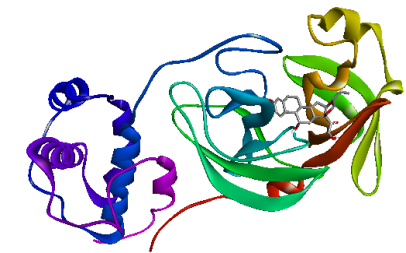 |
| 2 | Doxorubicin | 31703 | 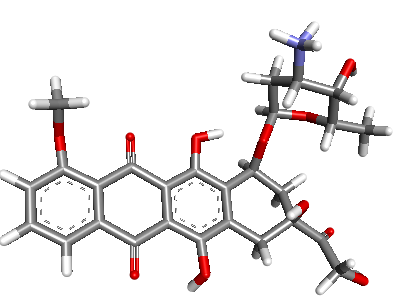 | 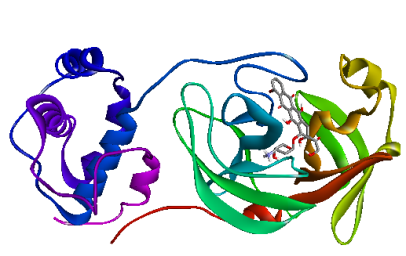 |
| 3 | Dexamethasone | 5743 | 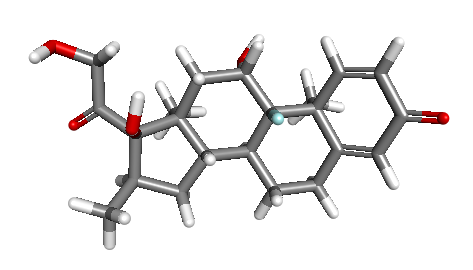 | 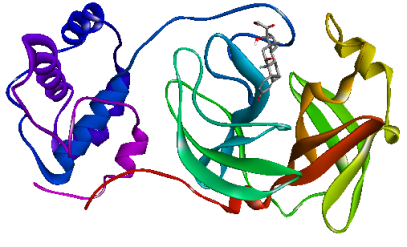 |
| 4 | Baricitinib | 44205240 | 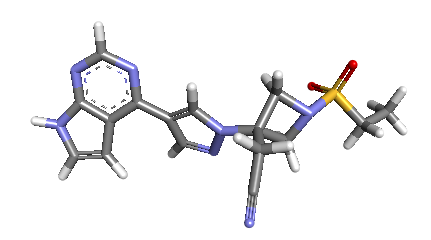 | 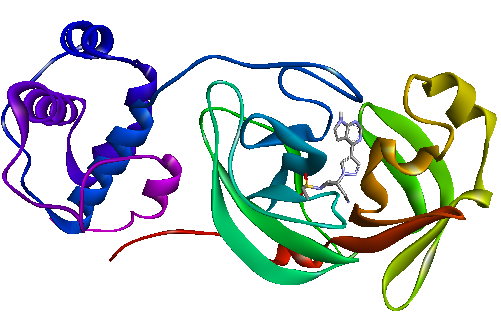 |
| 5 | Remdesivir | 121304016 | 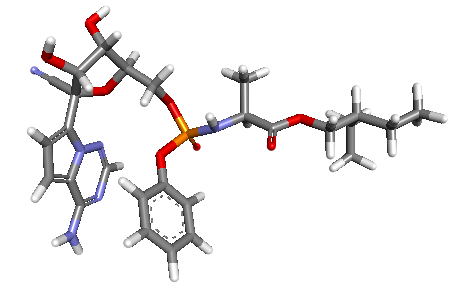 | 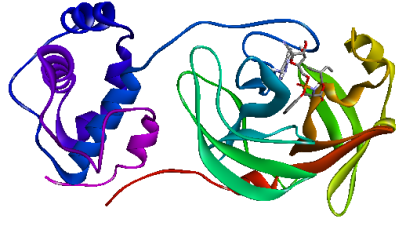 |
