# Supplementary material for: Discovery of new drug indications for COVID-19: A drug repurposing approach
Source: PLoS One. 2022 May 24;17(5):e0267095. doi: 10.1371/journal.pone.0267095 (PMC9129022; doi:10.1371/journal.pone.0267095)
Supplement: S1 Table — (DOCX) [file pone.0267095.s003.docx]

**Supplementary Table (S2) : List of Target genes for Covid-19**

| ABCC3 | ITGAV | ENO1 | VIM | SLC38A2 | RPL9 |
| --- | --- | --- | --- | --- | --- |
| ALDOA | LDHA | H1F0 | CFL1 | TNFAIP2 | SLC7A11 |
| CTGF | ACE2 | ITGB1 | ERBB2 | YBX1 | TOP2A |
| FASN | AMIGO2 | LIF | HMGA1 | CPLX2 | YWHAZ |
| HSP90AA1 | CXCL5 | ACTB | JUN | EREG | CRIM1 |
| KRT19 | FDCSP | ANTXR1 | LOXL2 | HNRNPA2B1 | FADS2 |
| MYL6 | HSP90AB1 | CYP24A1 | ACTG1 | KRT18 | MIEN1 |
| PMEPA1 | KRT23 | FDPS | ANXA2 | ACTN4 | STAT1 |
| RPL4 | NAMPT | HSPA8 | DCBLD2 | ASNS | AHNAK |
| SCD | PPIA | KRT5 | FLNB | DDIT4 | ASPH |
| TGM2 | RPL5 | NAP1L1 | ICAM1 | FTH1 | DHCR24 |
| UBC | SERPINA3 | PPP1R15A | KRT6A | IER3 | FTL |
| CALR | TIPARP | RPL7 | NEAT1 | KRT7 | IFIT2 |
| EIF5A | VEGFA | SERPINE1 | PSAT1 | NFKBIA | KRT8 |
| GSTP1 | CD24 | TMSB4X | RPL7A | PSMD3 | NPM1 |
| NQO1 | LAMB3 | ITGA2 | GPX2 | CLEC4M | CCL2 |
| RAI14 | OAS2 | LAMC2 | ITGA3 | CTSL | IFNA1 |
| RPS20 | RNF213 | P4HB | LCN2 | FURIN | CCR5 |
| SPP1 | RPS24 | RPL10 | PABPC1 | SH2D3A | CCL5 |
| TPM1 | SPTBN1 | RPS27 | RPL12 | CTRL | TMPRSS2 |
| ALDH3A1 | TPT1 | SQSTM1 | RPS27A | ICAM3 | AAK1 |
| PGK1 | PKM | TRAM1 | STARD3 | CXCL10 | CXCL8 |
| RPL26 | RPL28 | PLAU | TUBA1B | IL6 | CXCL9 |
| RPS4X | RPS6 | RPL3 | PLOD2 | CRP | GAK |
| TGFBI | SAT1 | S100A2 | RPL37 | IRF3 | MX1 |
| AKR1B1 | TXNIP | TXNRD1 | S100A6 | MBL2 | IFNG |
| ATP1B1 | AKR1B10 | TUBB | TUBB4B | TMPRSS11D | JAK1 |
| EEF2 | C3 | AKR1C2 | MTHFD2 | CD209 | DDX58 |
| GAPDH | EIF1 | CALM2 | TNF | IL10 | IFNB1 |
| IGFBP4 | GAS5 | EIF4B | CD40LG | GPT | IL2 |
| PTMA | RPS19 | SLC7A5 | TPI1 | ALDH1A1 | PFN1 |
| RPL23 | RPS3A | STC2 | AKAP12 | ATF4 | EEF1A1 |
| G6PD | IGFBP3 | KRT81 |  |  |  |
