# Supplementary material for: Discovery of new drug indications for COVID-19: A drug repurposing approach
Source: PLoS One. 2022 May 24;17(5):e0267095. doi: 10.1371/journal.pone.0267095 (PMC9129022; doi:10.1371/journal.pone.0267095)
Supplement: S2 Table — (DOCX) [file pone.0267095.s004.docx]

**Supplementary Table (S3): Molecular features used for Clustering**

| Heavy atoms | Hydrogen bond donors |
| --- | --- |
| Fraction.Csp3 | TPSA |
| Hydrogen bond acceptors | XLOGP3 |
| Molar Refractivity | MLOGP |
| iLOGP | Consensus.Log.P |
| WLOGP | ESOL.Solubility..mg.ml. |
| Silicos.IT.Log.P | ESOL.Class |
| ESOL.Log.S | Ali.Solubility..mg.ml. |
| ESOL.Solubility..mol.l | Ali.Class |
| Ali.Log.S | Silicos.IT.Solubility..mg.ml. |
| Ali.Solubility..mol.l | Silicos.IT.class |
| Silicos.IT.LogSw | BBB.permeant |
| Silicos.IT.Solubility..mol.l | CYP1A2.inhibitor |
| GI.absorption | CYP2C9.inhibitor |
| Pgp.substrate | CYP3A4.inhibitor |
| CYP2C19.inhibitor | Lipinski..violations |
| CYP2D6.inhibitor | Veber violations |
| log.Kp..cm.s. | Muegge violations |
| Ghose..violations | PAINS alerts |
| Egan..violations | Brenk..alerts |
| Bioavailability.Score | Synthetic.Accessibility |
| Aromatic.heavy.atoms | MW |
| Rotatable.bonds |  |
